# Active Poly(3-hydroxybutyrate-co-3-hydroxyvalerate) Electrospun Membranes Incorporating Antioxidant Oil from Spent Coffee Grounds

**DOI:** 10.3390/polym18212780

**Published:** 2026-11-07

**Authors:** Laura Vignau Arsuaga, Aloña Retegi, Irati Berasarte, Loli Martin, Arantxa Eceiza, Nagore Gabilondo

**Affiliations:** 1 “Materials + Technologies” Group (GMT), Department of Chemical and Environmental Engineering, Faculty of Engineering of Gipuzkoa, University of the Basque Country (UPV/EHU), 20018 Donostia-San Sebastián, Spain; laura.vignau@ehu.eus (L.V.A.); alona.retegui@ehu.eus (A.R.); irati.berasarte@ehu.eus (I.B.); 2 Macrobehavior-Mesostructure-Nanotechnology, General Research Service (SGIker), Faculty of Engineering of Gipuzkoa (GIE), University of the Basque Country, Plaza Europa 1, 20018 Donostia-San Sebastián, Spain; loli.martin@ehu.eus

**Keywords:** poly(3-hydroxybutyrate-co-3-hydroxyvalerate) (PHBV), electrospinning, nanofiber membranes, coffee oil, spent coffee grounds, antioxidant activity, coaxial electrospinning

## Abstract

Active poly(3-hydroxybutyrate-co-3-hydroxyvalerate) (PHBV) nanofiber membranes incorporating antioxidant extracted coffee oil (ECO) from spent coffee grounds were prepared by electrospinning. GC-MS analysis revealed similar proportions of saturated and unsaturated fatty acids, predominantly linoleic (37%) and palmitic (35%) acids, followed by oleic/elaidic (9%) and stearic (8%) acids. ECO exhibited antioxidant activity in DPPH and ABTS assays (82 and 51 μmol Trolox equivalents, respectively), with a total phenolic content of 22 mg gallic acid equivalents per gram. Single-needle electrospinning produced membranes containing 8–30 wt% ECO, while coaxial electrospinning of an ECO core and PHBV shell yielded membranes containing 15 wt% ECO. Thermal, morphological, surface, mechanical, and antioxidant properties were investigated. Single-needle electrospinning produced continuous, bead-free nanofibers, whereas TEM observations suggested a core–shell structure in coaxial fibers. ECO incorporation increased fiber diameter (465–586 nm for single-needle and 542–619 nm for coaxial fibers), promoted fiber junctions and surface wettability, and significantly improved mechanical strength, particularly at the highest ECO loading. All ECO-loaded membranes showed radical scavenging activity, with coaxially electrospun membranes exhibiting DPPH inhibition (44%) comparable to that of single-needle membranes containing twice the ECO loading. These findings support the potential of ECO-loaded PHBV nanofibers as active membranes.

## 1. Introduction

Plastics are lightweight, durable and resistant to corrosion, properties that have driven their extremely high use in recent decades [1,2,3]. However, their petroleum-based nature makes them highly persistent, leading to a severe global waste-accumulation problem [4,5,6]. In this aspect, microbial polymers, biopolymers produced by microorganisms, represent a promising and sustainable alternative to conventional plastics. These polymers can be obtained from renewable sources and degrade naturally, making them crucial to the development of bioplastics and advanced materials with applications in key fields such as biomedicine, food industry, and packaging. Polyhydroxyalkanoates (PHAs) are naturally occurring polyesters that many organisms accumulate within their cells to conserve oxygen and energy in the face of stress and nutrient deficiency [7,8,9]. Moreover, PHAs can, in fact, be processed for use in a wide range of products, such as films, adhesives, non-woven textiles, paper coatings, packaging and molded goods, so represent a sustainable alternative to conventional commodity plastics.

Among the different types of PHAs, poly(3-hydroxybutyrate-co-3-hydroxyvalerate) (PHBV) stands out as a promising material for numerous applications due to its thermo-plastics characteristics together with its inherent biocompatibility, biodegradability and non-toxicity [10]. In this context, PHBV is also being investigated for the fabrication of electrospun nanofibrous membranes for various applications, (packaging, drug delivery, agricultural releasing systems, tissue engineering and other medical applications) [11,12,13]. Electrospinning is a widely used and scalable technique for producing ultrathin continuous fibers with high surface-to-volume ratios and interconnected pores by applying a high-voltage electric field [14,15]. While conventional electrospinning is the most common approach, coaxial electrospinning has gained increasing attention [16]. This technique enables the simultaneous electrospinning of two solutions through concentrically aligned spinnerets, producing core–shell nanofibers [17]. Such structures combine the properties of different materials, providing mechanical stability while protecting encapsulated sensitive compounds from degradation and enabling their sustained release [18]. Yang et al. demonstrated that electrospinning is an efficient method to protect fish oil against oxidation, especially at high temperatures [19]. Their study showed that coaxial nanofibers provide better stability because the core–shell structure creates a double barrier that effectively blocks oxygen from reaching the oil.

Coffee is the second most consumed beverage worldwide after water, leading to the generation of large quantities of residues, mainly spent coffee grounds (SCG) and coffee husks. In 2021, global coffee consumption reached approximately 175,600 million bags (ICO, 2023) [20], resulting in the disposal of millions of kilograms of coffee waste each year. SCG contain a wide variety of bioactive compounds such as antioxidants, caffeine, and tannins, which confer significant potential for their valorization in high-added-value applications such as food, pharmaceutical, cosmetic, and sustainable packaging, representing a substantial, yet underutilized, valuable resource.

Although the incorporation of spent coffee grounds (SCG) into PHBV-based biocomposites has been previously investigated, most reported studies have focused on the use of SCG as solid biofillers in materials processed by conventional techniques, such as extrusion and compression molding [21,22]. Alternatively, several studies have explored the incorporation of SCG-derived extracts into polymeric films, particularly for the development of active food packaging materials with enhanced antioxidant and functional properties [23,24]. Papadaki et al. [25] prepared whey protein edible films and observed an improvement in the hydrophobic and antioxidant properties of the films with the incorporation of oils and derivatives extracted from SCG. However, comparatively fewer studies have investigated the incorporation of these extracts into electrospun nanofibers. In this context, two studies reported the incorporation of SCG extracts into zein-based electrospun matrices [26,27]. Doostan et al. [28] developed PVA/PCL nanofibers loaded with 10 wt% coffee and calendula extracts, achieving high antioxidant activity and better cell migration. Despite these advances, the use of PHBV as a polymeric matrix for the incorporation of SCG-derived extracts remains largely unexplored, particularly in electrospun systems. To the best of our knowledge, the incorporation of SCG-derived extracts into PHBV nanofibers using a coaxial electrospinning configuration has not yet been reported. Therefore, the present study explores a promising strategy for the valorization of coffee waste through the development of PHBV-based core–shell nanofibrous membranes.

This research focuses on the development of electrospun bioactive nanofiber membranes of PHBV loaded with extracted coffee oil (ECO) from SCG, employing both single and coaxial electrospinning configurations. The obtained membranes were thoroughly characterized in terms of their ECO loading capacity and bioactivity, as well as mechanical, physicochemical and morphological/surface properties. The combined use of SCG-derived bioactive compounds and advanced electrospinning strategies led to functional PHBV-based materials, with the potential to enhance nanofiber membrane properties and enable their application in a wide range of high-performance systems.

## 2. Materials and Methods

### 2.1. Materials

Commercial PHBV (ENMAT Y1000P PHBV, Tianan Biologic Materials, Ningbi, China) with 2–3 mol% 3-hydroxyvalerate (3HV) according to the data sheet from manufacturer, was provided by Helian Polymers BV, Netherlands. Trifluoroethanol (TFE, ≥99% purity) was supplied by Merck (Darmstadt, Germany). Dimethyl sulfoxide (DMSO, ≥99.9% purity), dichloromethane (DCM, ≥99.8% purity), acetone (≥99.5% purity), methanol (≥ 99.9% purity), sodium hydroxide (NaOH, pellets) were purchased from Sigma-Aldrich(Darmstadt, Germany). The reagents for the determination of antioxidant capacity, i.e., 2,2-diphenyl-1-picrylhydrazyl (DPPH), (2,2′-azino-bis(3-ethylbenzothiazoline-6-sulfonic acid) diammonium salt (ABTS), potassium persulphate (K_2_S_2_O_8_), Folin-Ciocalteau reagent, and sodium carbonate (Na_2_CO_3_), and 6-hydroxy-2,5,7,8-tetramethylchroman-2-carboxylic acid (Trolox) and gallic acid standards, were purchased from Sigma-Aldrich.

### 2.2. Methods

#### 2.2.1. Extraction of ECO

SCG of medium-roasted *Coffea arabica* coffee beans roasted by a local coffee shop were used as raw material to obtain the ECO. SCG were thoroughly washed with deionized water at room temperature to remove potential contaminants and water-soluble compounds. Washing was continued until a neutral pH was reached. The washed SCG were subsequently oven-dried at 110 °C overnight, and ground for 5 min using a commercial grinder. For the extraction procedure, 20 g of dried ground SCG were mixed with 100 mL of acetone in a round bottom flask equipped with a magnetic stirrer. The mixture was heated under reflux at 60 °C for 24 h while stirring at 400 rpm. After this period, the solid residue was separated from the liquid extract by vacuum filtration: The acetone was removed from the filtrate by rotary evaporation to recover the extracted coffee oil (ECO), which was collected and stored in sealed vials at −20 °C until further analysis.

The extract yield was calculated as the mass of recovered extract relative to the initial mass of dried SCG. Approximately 2 g of ECO were obtained from 20 g of SCG, corresponding to an apparent extraction yield of 10 wt%. This yield was consistent with values typically reported for acetone extraction [29].

#### 2.2.2. Membranes Fabrication by Electrospinning

Fluidnatek LE-10 lab equipment (Bioinicia S.L.,Valencia, Spain) with both single and coaxial needle configurations was used. Neat PHBV nanofibers were electrospun as a control in both single and coaxial configurations. For single electrospinning, a solution was prepared by dissolving 1.50× *g* of PHBV in 8 mL TFE (density 1.373 g/mL) at 50 °C through gentle stirring overnight to produce a PHBV solution in TFE of 12 wt%. The solution was electrospun using a constant flow rate of 2 mL/h, a needle tip to the collector distance of 28.5 cm, a voltage range of 8.8–11.5 kV and a disposable Luer Lock needle (18G-½″) as the spinneret. For coaxial electrospinning, the solution of 12 wt% PHBV in TFE was used as the outer electrospinning solution to form the shell of hollow nanofibers. The coaxial electrospinning parameters were a flow rate of 2 mL/h, a voltage of 11–11.5 kV, and a distance of 28.5 cm between the needle tip and the collector. A coaxial spinneret composed of two concentric needles was employed. The outer needle was a disposable Luer Lock (16G-½″ (black)), and the inner needle was a stainless steel capillary needle of 63 × 0.9 × 0.6 mm (length and outer and inner diameters, respectively). Single and coaxially produced PHBV neat nanofiber membranes were labelled as SPHBV and CPHBV, respectively.

Active nanofibers loaded with ECO were prepared by single and coaxial electrospinning. For the single configuration, three PHBV solutions (12 wt% in TFE) were loaded with 1, 3 or 5 wt% ECO, respectively, and subsequently electrospun using the same parameters as those employed for neat PHBV. The theoretical final ECO content of the PHBV-based nanofiber membranes after solvent evaporation was 8, 20 and 30 wt%, respectively. In the coaxial configuration, neat ECO was used as the inner fluid forming the core of the nanofiber, while the outer solution consisted of the same PHBV in TFE (12 wt%) forming the nanofiber shell. Different flow rates of 2 mL/h and 0.05 mL/h were used for the outer PHBV solution and the inner ECO fluid, respectively. The applied voltage ranged from 11 to 11.5 kV and the distance between the needle tip and the collector was 28.5 cm. Considering the density values calculated for the PHBV solution and the ECO as 1.33 ± 0.09 g/mL and 1.09 ± 0.12 g/mL, respectively, the theoretical ECO content in the coaxial nanofiber membranes was estimated to be 15 wt%.

Both single and coaxial membranes were produced by collecting electrospun nanofibers on aluminum foil for 2 h, yielding membranes with dimensions of 20 × 20 cm and a thickness of 120 ± 25 µm. Active membranes were labelled “SXPHBV” or “CXPHBV” according to the single (S) or coaxial (C) electrospinning configuration employed, respectively, and the theoretical ECO content (X). In summary, unloaded membranes (SPHBV and CPHBV) and ECO-loaded membranes (S8PHBV, S20PHBV, S30PHBV and C15PHBV) were produced and stored in a desiccator before being characterized.

#### 2.2.3. Characterization of the Antioxidant Activity of ECO

To evaluate the antioxidant properties of the ECO, three assays were employed: the 2,2-diphenyl-1-picrylhydrazyl (DPPH) free radical scavenging capacity assay, the 2,2′-azinobis(3-ethylbenzothiazoline-6-sulfonic acid (ABTS) radical cation decoloration assay, and the determination of total phenolic content (TPC).

The DPPH assay was carried out in accordance with the protocol established by Payum et al. [30]. Briefly, 2.9 mL of DPPH reagent (0.06 mM in MeOH) was added to 100 μL of sample solution (2.8 mg/mL ECO in MeOH), or Trolox standard, and thoroughly mixed. DPPH decoloration was measured at 517 nm using a UV-Vis spectrophotometer after the reaction mixture was left in the dark for 30 min at room temperature. A linear calibration curve was established using Trolox standard (R^2^ = 0.9905) (Figure S1), and antioxidant capacity was expressed as Trolox equivalents per gram of dry sample.

The ABTS assay was performed, with minor adjustments, in accordance with Payum et al., [30]. The ABTS solution (7 mM) was oxidized by reacting with potassium persulfate (2.45 mM aqueous solution) and incubated in the dark at room temperature for 12–16 h to generate the ABTS•^+^ radical cation, resulting in a characteristic dark green coloration. Antioxidant activity, based on free radical scavenging capacity, was evaluated by mixing 50 µL of sample extract (2.8 mg/mL ECO in MeOH), or Trolox standard, with 1 mL of the ABTS•^+^ working solution in a microcuvette. Absorbance was recorded using an UV-Vis spectrophotometer at 734 nm, exactly 1 min after mixing, followed by a final measurement at minute 6, with the final absorbance used for analysis. A linear calibration curve was established using Trolox standard (R^2^ = 0.9987) (Figure S2), and antioxidant capacity was expressed as Trolox equivalents per gram of dry sample.

The TPC of ECO was determined using the Folin–Ciocalteu (FC) procedure, according to Figueira et al. [31], with a few modifications. Briefly, 1 mL of sample extract (2.8 mg/mL ECO in MeOH) was mixed with 0.5 mL of FC reagent, and after 5 min, 1.6 mL of Na_2_CO_3_ (7.5 g/100 mL aqueous solution) and 7.1 mL of distilled water were added to finish the reaction. The reaction mixture was kept in the dark at room temperature for 2 h, after which the absorbance was measured using an UV-Vis spectrophotometer at a wavelength of 765 nm. A standard calibration curve was prepared using aqueous gallic acid solutions (n = 8) ranging from 1 mg/L to 8 mg/L, and reacted with FC reagent (R^2^ = 0.9965) (Figure S3). The total phenolic content was expressed as gallic acid equivalent per gram of dry sample.

### 2.3. Characterization Techniques

The composition of ECO was analyzed by Gas Chromatography coupled to a mass spectrometer (GC-MS; 6890 Network GC coupled to a 5973 mass spectrometer, equipped with a 7693 autosampler, Agilent Technologies, Inc., Santa Clara, CA, USA). For the analysis, the samples were esterified and extracted with organic solvent. Briefly, approximately 10 mg (0.01 g) of the sample was accurately weighed using an analytical balance and transferred to a Pyrex digestion tube. Subsequently, 1.5 mL of 0.5 M NaOH in methanol was added, and the mixture was heated at 100 °C for 8 min in a digestion block. After this step, 2 mL of 15% H_2_SO_4_ in methanol was added, and the mixture was maintained at 100 °C for 1 h. The solution was then allowed to cool to room temperature, and 5 mL of saturated NaCl solution was added. Finally, extraction was performed using 1 mL of dichloromethane. The analyses were performed in SCAN mode using split injection (1:50). Each sample was injected in triplicate (n = 3), together with a standard control sample (37 fatty acid methyl esters (FAMEs; SRM 47885) and a blank sample. NIST14 library was also used for the identification of other compounds.

The density values of ECO and that of the PHBV solution in TFE (12 wt%) were determined by dividing their mass by their volume. A volume of 0.2 mL of each liquid was taken with a calibrated micropipette and added to an empty flask with known weight, and weighted in a precision balance. Measurements were performed in triplicate.

The thermal stability of the electrospun membranes and the ECO was examined by thermogravimetric analysis (TGA) using a TGA/DSC3+ Mettler Toledo instrument (Mettler Toledo, Switzerland) Measurements were performed using 5– 6 mg of sample and heating from 25 to 800 °C in a nitrogen atmosphere at a constant heating rate of 10 °C min^−1^. The maximum degradation temperature was calculated as the minimum of the degradation peak in the weight derivative curve.

Differential scanning calorimetry (DSC) was used to evaluate the thermal behavior of both the electrospun membranes and ECO. Measurements were carried out using a DSC3+ Mettler Toledo instrument (Mettler-Toledo GmbH, Greifensee, Switzerland). Approximately 6 mg of sample was placed in a closed aluminum capsule and heated from −70 to 250 °C at a heating rate of 10 °C min^−1^ under a nitrogen atmosphere at a 50 mL min^−1^ flow rate. The melting temperature was determined as the maximum of the endothermic peak and the melting enthalpy as the area of the peak.

The chemical structure of the membranes and the ECO was examined using Fourier transform infrared spectroscopy (FTIR). Spectra were recorded with a PerkinElmer Spectrum Two spectrometer equipped with a GladiATR™ accessory (PerkinElmer, Inc., Shelton, CT, USA) containing a monolithic diamond ATR crystal with temperature control (PIKE Technologies, Madison, WI, USA). Measurements were collected in the range of 650–4000 cm^−1^, averaging 32 scans at a resolution of 4 cm^−1^.

The morphology of the electrospun membranes was characterized using scanning electron microscopy (SEM) and transmission electron microscopy (TEM) to analyze fiber structure and diameter distribution. SEM imaging was carried out using a Hitachi S-4800 (Hitachi High-Technologies Corporation, Tokyo, Japan) microscope operating at 5 kV to observe the surface morphology of the nanofiber membranes. Before observation, the specimens were sputter-coated with an approximately 15 nm thick gold layer using an Emitech K550X sputter coater - (Quorum Technologies Ltd., Laughton, East Sussex, UK) to improve surface conductivity and enhance image resolution. The fiber diameter distribution of each electrospun membrane was subsequently determined using ImageJ software(v1.53e). Hitachi TM3030Plus microscope (Hitachi High-Technologies Corporation, Tokyo, Japan) was used for the acquisition of cross-sectional images, operating in standard mode at 15 KV. The samples were gold-coated using an Emscope SC 500 sputter coater (Emscope Laboratories Ltd., Ashford, Kent, UK).

TEM analysis was performed using a TECNAI G2 20Twin microscope (FEI Company, Hillsboro, OR, USA) operating at 200 kV to visualize the structure of the coaxial nanofibers (FEI, Eindhoven, The Netherlands). For analysis, the membrane was placed on 200-mesh carbon grids.

The surface properties of electrospun nanofiber membranes were evaluated by means of static water contact angle (WCA) measurements using a SEO-Phoenix-300-Touch instrument (Surface Electro Optics Co., Ltd., Suwon, Gyeonggi-do, Republic of Korea). A drop of 4 μL of deionized water was deposited on the membrane surface, and the contact angle value was calculated by averaging six measurements.

The mechanical behavior of the membranes was analyzed by means of tensile tests using an Instron 5967 universal testing machine (Norwood, MA, USA) equipped with a 500 N load cell. Rectangular shaped samples with dimensions of 30 × 6 mm were cut from the membranes, and clamped between pneumatic grips with a gauge length of 10 mm. The tests were performed at 2 mm min^−1^. Ultimate tensile strength (MPa) and elongation at break (%) were determined by averaging six measurements of each membrane.

The antioxidant capacity of ECO and the electrospun membranes was studied using UV-Vis spectrophotometry (Shimadzu UV-3600 UV-Vis-NIR spectrophotometer, Shimadzu Corporation, Kyoto, Japan). For the neat ECO, the DPPH, ABTS and TPC assays were performed as explained, whereas the antioxidant activity of the membranes was evaluated in terms of DPPH scavenging activity percentage.

For electrospun membranes, 10 mg of each sample were dispersed in a solvent mixture of 1 mL of DMSO and 1 mL of chloroform, and sonicated for 10 min. Then, 8 mL of methanol were added, and the mixture was sonicated for 10 min, allowing the nanofibers to sediment and leaving the antioxidant extract in the supernatant. The concentration of ECO in the supernatant was determined using properly prepared calibration curve (Figure S4). 1 mL of the supernatant was mixed with 2.9 mL of freshly prepared DPPH solution (0.06 mM in MeOH) and kept at room temperature in the dark. The absorbance at 517 nm was measured after 30 min. All measurements were performed in triplicate. The inhibition percentage was calculated using the following equation:

(1) $$\mathrm{DPPH}\;\mathrm{scavenging}\;\mathrm{activity}\;\%=\frac{{\mathrm{Abs}}_{\mathrm{DPPH}}-{\mathrm{Abs}}_{S}}{{\mathrm{Abs}}_{\mathrm{DPPH}}}\times 100,$$

where Abs_DPPH_ is the absorbance value at 517 nm of the control solution of DPPH in methanol and Abs_s_ is the absorbance value at 517 nm of the DPPH assay solution.

### 2.4. Statistical Analysis

Statistical analyses were performed using one-way analysis of variance (ANOVA) to evaluate differences among the PHBV-based membrane formulations. When comparing the different membrane formulations, Tukey’s test was used for pairwise comparisons. Statistical significance was set at *p* < 0.05.

The analyses of fiber diameters measured by SEM were performed using 50 measurements per formulation (n = 50). Water contact angle (WCA), tensile strength, and strain at break included six measurements per formulation (n = 6). The analyses of the amount of extracted oil and membrane antioxidant response measured by the DPPH assay included three observations per formulation (n = 3). Results are reported as mean ± standard deviation (SD).

## 3. Results and Discussion

### 3.1. Compositional Analysis and Antioxidant Capacity of ECO

The composition of the ECO was analyzed using GC-MS. The obtained chromatogram is presented in the Supplementary Material as Figure S5. The predominant fatty acids identified were palmitic acid and linoleic acid, accounting for 35.14% and 37.17, respectively. The other fatty acids identified were oleic acid and elaidic acid (*cis* and *trans* C18:1 isomers, respectively; combined relative abundance of 9.10%), stearic acid (8.28%), arachidic acid (3.49%), linolenic acid (1.88%), and behenic acid (1.17%). The results are consistent with the reported literature, even though the composition depends on the characteristics of the coffee waste and the extraction conditions. Leow et al. [29] characterized coffee oil using different organic solvents, also identifying palmitic, linoleic, stearic, and oleic acids as major constituents, together with caffeine and tocopherols. In our case, since the analysis focused on the identification and semiquantitative characterization of fatty acids, the main components of coffee oil, signals from other compounds could not be reliably confirmed under the analytical conditions employed.

The antioxidant capacity of the coffee extract was characterized by DPPH, ABTS and TPC assays. The results obtained are presented in Table 1.

The obtained results fall within the previously reported range for coffee waste derived compounds. As stated previously, previous studies report that SCG exhibit notable antioxidant potential, with values varying between approximately 75 and 170 µmol Trolox equivalents per gram of dry material [32,33]. Additionally, the TPC of this residual material was found to be in the range of 20–30 mg gallic acid equivalents per gram of dry waste [34]. Hwang et al. [35] investigated the phenolic fraction and reported TPC values close to 50 mg GAE/g extract for coffee extract obtained from acetone extraction, higher than the obtained in our study, but lower than that measured for more polar solvents. These findings suggest that the ECO obtained in the present study may contain a similar combination of lipid components and bioactive compounds.

According to the results obtained, the coffee extract obtained could be considered suitable for its use as an active component in the development of new materials intended for applications requiring antioxidant activity.

### 3.2. Thermal and Physicochemical Characterization of Electrospun Nanofiber Membranes

**Figure 1:**
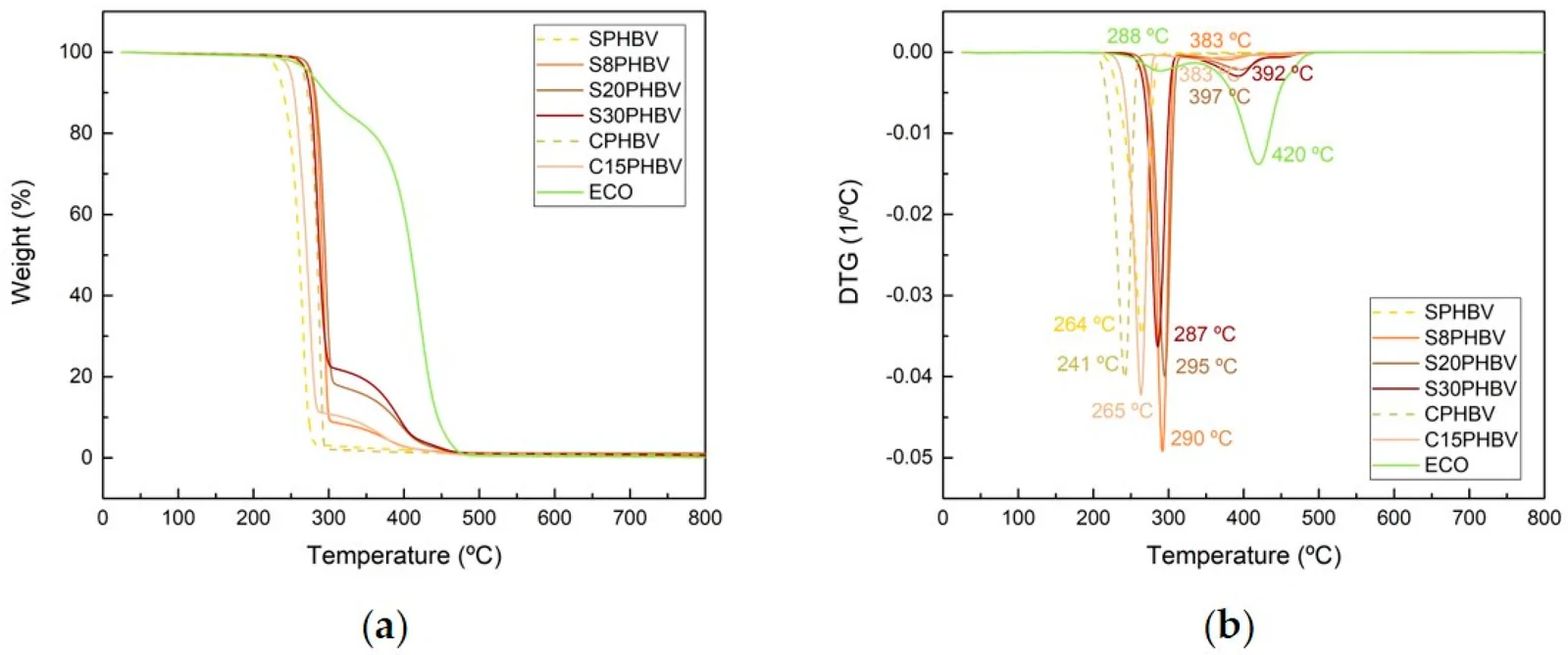
TG (**a**) and DTG (**b**) curves of ECO and PHBV-based nanofiber membranes obtained by single and coaxial electrospinning, with and without ECO (SPHBV, S8PHBV, S20PHBV, S30PHBV, CPHBV and C15PHBV).

The thermal stability of the PHBV-based nanofiber membranes was evaluated using thermogravimetric analysis. Figure 1a,b shows the weight evolution with temperature (TG) and their derivative (DTG) curves, respectively, of the electrospun membranes and of the pure ECO.

Regarding the degradation profile of neat PHBV nanofiber membranes, SPHBV and CPHBV, a slight difference in the degradation profile was observed between single and coaxial configuration. The SPHBV membrane degradation temperature was measured at 264 °C, whereas the CPHBV membrane was 241 °C, suggesting that nanofiber configuration influenced their thermal stability. Previous studies on coaxial electrospun nanofibers reported that the addition of a core component often lowers the thermal degradation compared to one component nanofibers, likely due to the presence of internal core–shell interfaces and heterogeneous domains that act as weaker regions for decomposition during heating [36]. In our case, even if a second component does not exist in the case of the hollow nanofiber, it could still exhibit the same pattern.

For ECO, the thermogravimetric profile showed an initial degradation step with a weight loss of around 16% occurring between approximately 230 °C and 320 °C, followed by a second, higher and broader decomposition stage extending from around 320 °C up to 500 °C with a weight loss of around 80% (Figure S6). These temperature intervals were consistent with the behavior reported by Raba et al. [37] for green coffee bean oil, who observed two main degradation regions between 220 and 365 °C and 340–525 °C. The first one, which was smaller, attributed to the decomposition of unsaturated acids, while the second one, the major mass loss, was primarily associated with the thermal breakdown of saturated fatty acids and the unsaturated acids fraction remaining from the first step, were influenced by composition and thermal treatment of coffee. Concerning ECO-loaded electrospun membranes, the TG curves of single and coaxial PHBV-based nanofiber membranes loaded with different ECO contents showed two well-defined degradation stages. The first stage, which occurred at temperatures between 250 and 280 °C, was primarily attributed to PHBV. However, it also coincided with the initial weight loss observed for neat ECO. The second stage, which occurred at higher temperatures, corresponded to the thermal decomposition of the more stable ECO fraction. Analysis of the degradation profiles of membranes with varying ECO content reveals that thermal stability increased with ECO incorporation, for both single- and coaxial-electrospun membranes. The maximum degradation temperature shifts from 264 °C for the SPHBV membrane to 290, 295 and 287 °C for the S8PHBV, S20PHBV and S30PHBV membranes, respectively, and from 241 °C for the CPHBV membrane to 290 °C for the C15PHBV membrane. Overall, these results suggest that incorporating ECO improves the thermal stability of PHBV by delaying its degradation at higher temperatures.

Analysis of the degradation profile of membranes also shows that mass loss associated with the second stage of ECO degradation increased with extract content in the membrane. The difference in mass loss between loaded and unloaded membranes at this interval, referred to the ECO weight loss in the second interval, can be used to experimentally confirm the ECO content of the membranes. The obtained values reveal an ECO content of 10%, 21% and 32% in membranes S8PHBV, S20PHBV and S30PHBV, and 14% in membrane C15PHBV (Figure S7). Despite the overlap of the slight mass loss experienced by PHBV within these degradation interval, these values are close to the theoretical ECO content of the membranes.

**Figure 2:**
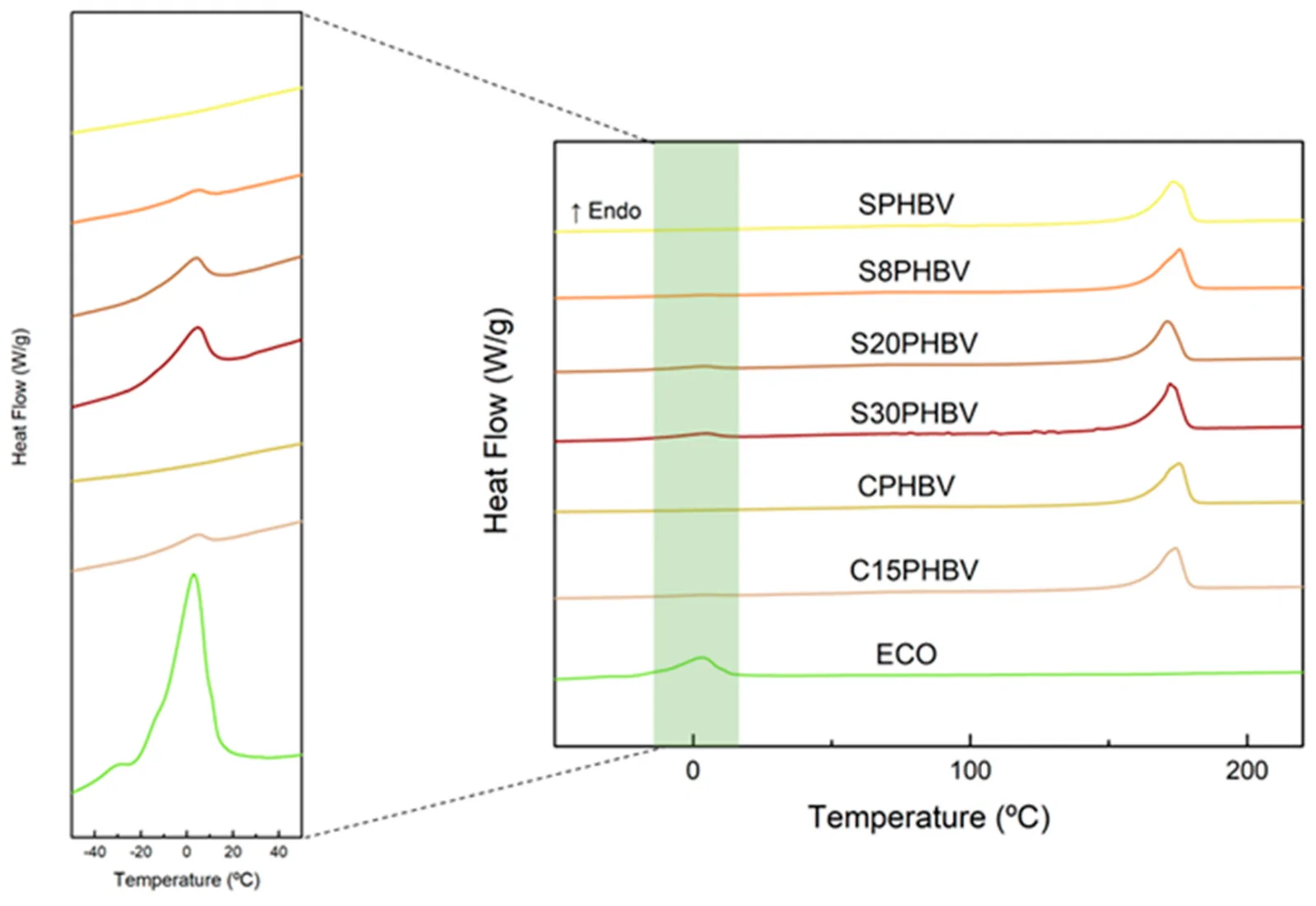
DSC thermograms of ECO and PHBV-based nanofiber membranes obtained by single and coaxial electrospinning, with and without ECO (SPHBV, S8PHBV, S20PHBV, S30PHBV, CPHBV and C15PHBV).

Figure 2 shows the DSC thermograms of the neat PHBV membranes, SPHBV and CPHBV, ECO, and ECO-loaded electrospun PHBV-based nanofiber membranes, S8PHBV, S20PHBV, S30PHBV and C15PHBV.

As can be observed, SPHBV and CPHBV exhibited a single endothermic peak at approximately 170 °C, corresponding to the melting transition of the polymer. A slightly lower melting enthalpy was measured for CPHBV than for SPHBV (56.5 J/g vs. 60.3 J/g), suggesting that the spinning configuration influenced polymeric chain mobility arrangement. Regarding the coffee extract, an endothermic transition was observed near 5 °C, which was attributed to the melting of its saturated fatty acids [38]. The membranes loaded with ECO showed the characteristic melting transition of both ECO and PHBV within the same temperature ranges. The enthalpy values of these transitions increased according to the content of each component. Indeed, ECO-loaded membranes showed a lower melting enthalpy value at 170 °C than unloaded membranes, in both single and coaxial configurations. Similarly, the melting enthalpy associated with ECO was also observed in the S8PHBV, S20PHBV, S30PHBV and C15PHBV membranes and increased with the ECO content (Table S1). Regarding the measured enthalpy values, and considering the melting enthalpy of neat ECO, the results suggest that a fraction of the oil incorporated into the nanofibers may remain non-crystalline, possibly because it is dispersed among the polymeric chains that could hinder its crystallization. The lower crystallinity measured in coaxially encapsulated ECO could be associated with both impediments for the crystallization of the oil in the core and heat transmission issues related to the encapsulation in core–shell morphology [39,40,41]. Feng et al. [41] attributed to chain mobility restrictions the limited crystallization of poly(ethylene glycol) confined in core–shell-like fibers. However, it is important to highlight that the preservation of the characteristic melting behavior of the coffee oil and PHBV suggests that both the oil and the PHBV retained their structural integrity and low degree of mixing throughout the electrospinning process.

### 3.3. FTIR Characterization of PHBV Nanofiber Membranes and ECO

**Figure 3:**
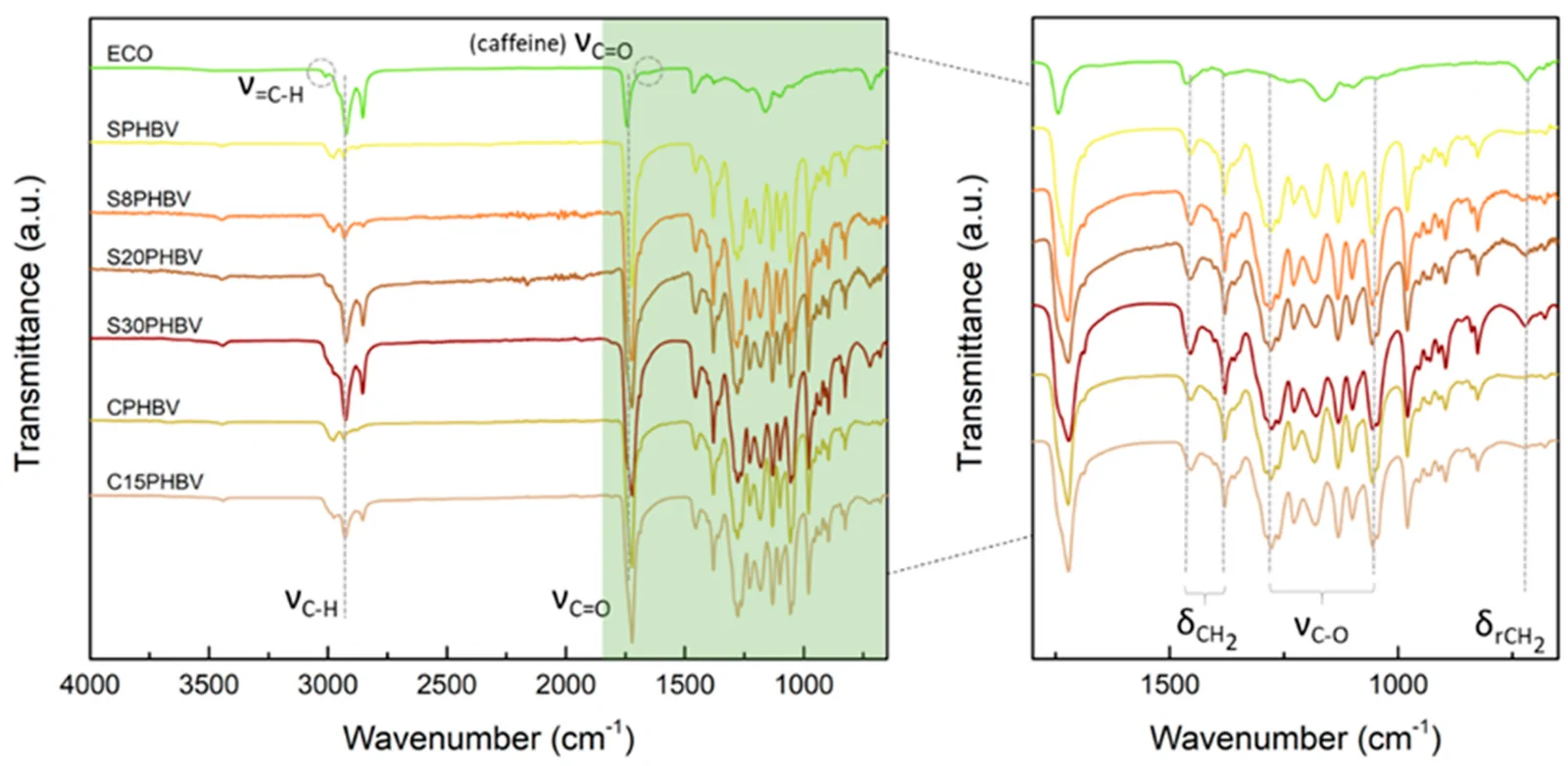
FTIR spectra of ECO and PHBV-based nanofiber membranes obtained by single and coaxial electrospinning configurations, with and without ECO (SPHBV, S8PHBV, S20PHBV, S30PHBV, CPHBV and C15PHBV).

The structural characterization of PHBV-based nanofiber membranes obtained by single and coaxial electrospinning, with and without ECO, as well as of ECO, was performed using FTIR spectroscopy and the obtained spectra were presented in Figure 3 and Figure S8.

As could be observed, the spectra of neat PHBV-based membranes, SPHBV and CPHBV, were very similar. Both samples showed characteristic bands of aliphatic polyesters at 2935 cm^−1^ and 2979 cm^−1^, which can be attributed to symmetric and asymmetric stretching vibrations of the C-H bond [42]. Bands associated with bending vibration of the C-H bond were also observed at 1450 and 1380 cm^−1^. The spectra also showed bands characteristic of the ester groups, with the prominent band indicative of the stretching vibration of the C=O bond at 1720 cm^−1^, as well as the stretching vibration bands of the C-O bond at 1280 and 1050 cm^−1^.

The FTIR spectrum of ECO revealed a broad band in the 3500–3200 cm^−1^ interval, which was attributed to the O-H stretching vibrations in intra- and intermolecular hydrogen bonding involving alcohols, phenols, and carboxylic acids (Figure S8). Moreover, the small band observed at 3008 cm^−1^, attributed to the =C-H stretching vibration, confirmed the presence of unsaturated fatty acids, mainly linoleic and oleic/elaidic acids, and polyphenols in the coffee extract. The distinctive band of the caffeine component of ECO was evidenced by a very small band that appeared at around 1650 cm^−1^, which was attributed to the C=O stretching vibration. This vibration band was used to estimate the caffeine content of standards and samples [43].

Moreover, ECO exhibited vibrational modes consistent with the presence of triglycerides and long-chain saturated fatty acids. The typical FTIR bands of triglycerides and long-chain saturated fatty acids, mainly palmitic and stearic acids, appeared at 2920 and 2850 cm^−1^ (C–H stretching), 1740 cm^−1^ (C=O stretching), 1460 cm^−1^ (CH_2_ bending) and 720 cm^−1^ (CH_2_ rocking). These vibrational modes were consistent with the FTIR profiles reported for coffee oil, in which triglycerides and saturated fatty acids exhibit the same characteristic bands [44]. Therefore, their presence in ECO is coherent with the known lipid fraction of coffee by-products, and complements the signals related to polyphenol and caffeine described above.

The FTIR spectra of PHBV-based nanofiber membranes loaded with ECO mainly showed the characteristic bands of PHBV as the main component. However, additional signals attributed to ECO were also distinguished. A distinct double band, which was already observed in the coffee extract at 2829–2882 cm^−1^, became more pronounced as the ECO content increased, confirming the incorporation of ECO into the PHBV-based nanofiber membranes. A similar trend was observed for the band at 1720 cm^−1^, which is characteristic of C=O stretching. This band slightly broadened and also became more pronounced as the ECO content increased. Finally, an increase in the intensity of the CH_2_ rocking band (δᵣCH_2_) at 720 cm^−1^ was also observed with increasing ECO content, which can be attributed to the greater contribution of aliphatic compounds present in the extract.

The FTIR spectra did not reveal clear evidence of remarkable chemical interactions between the polymer and the oil, as the characteristic absorption bands remained at their expected positions, without noticeable shifts, broadening, or the appearance of new signals. These observations were consistent with the thermal analyses, as neither DSC nor TGA revealed substantial changes that could be directly associated with interactions between the components. Taken together, the FTIR, DSC, and TGA results suggest that the incorporation of ECO did not induce major structural or chemical modifications in the polymeric matrix. Nevertheless, the occurrence of weak intermolecular interactions between the components cannot be ruled out based on these results.

### 3.4. Morphological, Superficial and Mechanical Properties

**Figure 4:**
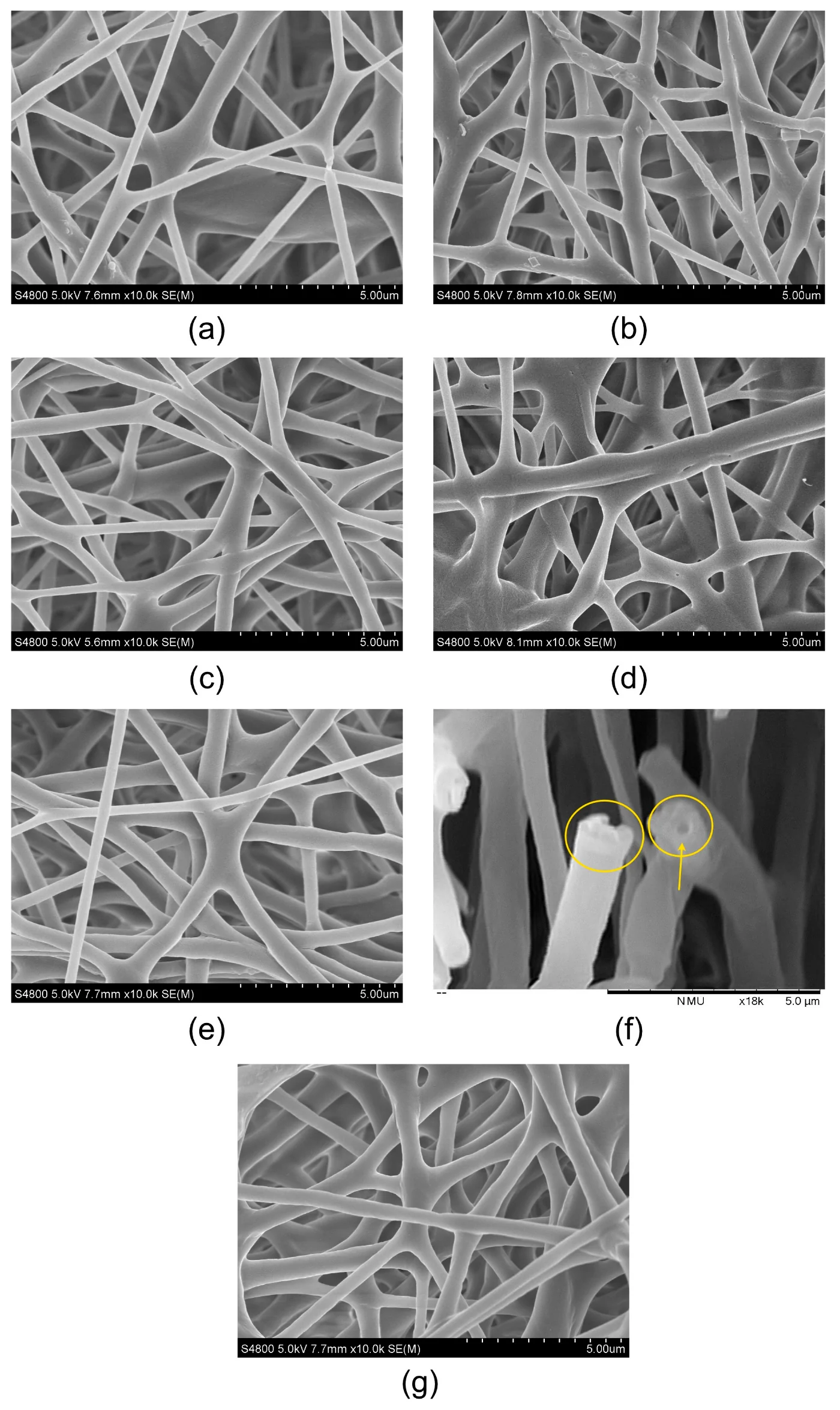
SEMimages of PHBV-based nanofiber membranes obtained by single and coaxial configuration: (**a**) SPHBV; (**b**) S8PHBV; (**c**) S20PHBV; (**d**) S30PHBV; (**e**) CPHBV; (**f**) cross-sectional CPHBV (yellow circles show cross-section of fractured nanofibers; arrow highlights the hollow core); (**g**) C15PHBV.

The morphology of the prepared PHBV-based nanofiber membranes obtained by single and coaxial electrospinning, and with and without ECO, was analyzed by SEM (Figure 4).

As it could be seen in SEM images, the neat SPHBV and CPHBV membranes and those loaded with ECO (S8PHBV, S20PHBV, S30PHBV, and C15PHBV) showed adequate spinnability, both by single and coaxial electrospinning, resulting in morphologically homogeneous nanofibers with diameters in the range of 465–619 nm. The corresponding diameter distribution histograms are presented in the SI (Figure S9). When comparing the diameters of the nanofibers loaded and unloaded with coffee extract, an increase in nanofiber diameter was observed with the presence of the extract. The mean diameter of the nanofibers in the membranes obtained by single electrospinning configuration was 465 ± 157 nm for SPHBV, increasing to 493 ± 238 nm, 501 ± 138 nm, and 586 ± 194 nm for S8PHBV, S20PHBV and S30PHBV, respectively, exhibiting significant differences (one-way ANOVA, F(5, 294) = 5.45, *p* < 0.001). The post hoc analysis showed that S30PHBV exhibited significantly larger fiber diameters than SPHBV (*p* = 0.011). Likewise, C15PHBV presented significantly larger fiber diameters than SPHBV (*p* < 0.001), S8PHBV (*p* = 0.006), and S20PHBV (*p* = 0.014). Kaniuk et al. [45], reported the same increase in diameter for nanofibers prepared from a PHBV solution loaded with different amounts of evening primrose oil. In addition to the observed increase in diameter with increasing oil content, SEM image of S30PHBV nanofiber membrane showed regions of interfiber junctions, suggesting that partial coalescence of the nanofibers occurred with increasing oil content. Regarding coaxially prepared nanofiber membranes, a similar diameter increase trend was observed, with mean diameter values of 542 ± 175 nm and 619 ± 152 nm for the neat CPHBV and ECO-loaded C15PHBV nanofibers, respectively. Notably, the coaxially prepared nanofibers, both unloaded and loaded with coffee extract, exhibited larger diameters than those prepared by single spinning. When comparing unloaded SPHBV and CPHBV nanofibers, the latter exhibited a larger diameter despite the inner channel not containing any solution. This difference in diameter between single and coaxially obtained nanofibers may mainly be due to the larger outer diameter of the coaxial needle. Furthermore, in the absence of oil, in hollow CPHBV nanofiber membranes, the unsupported core could collapse during solvent evaporation, producing flatter and smoother fibers in some regions. However, cross-sectional SEM image (Figure 4f) revealed hollow structure in the nanofibers. These morphological differences further support the idea that the oily phase acts as the core and influences how the polymeric shell is organized during electrospinning.

**Figure 5:**
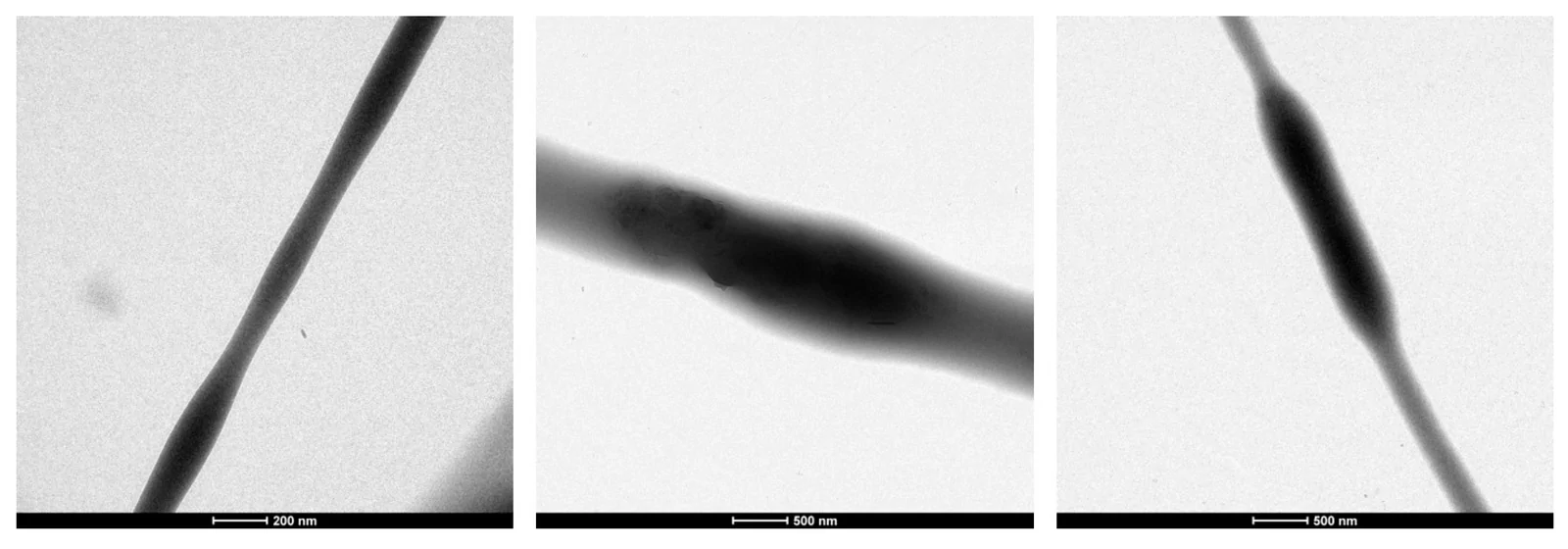
TEM images of PHBV-based nanofibers loaded with ECO and obtained by coaxial electrospinning (C15PHBV).

In order to investigate the distribution of the oil within the nanofiber, the ECO-loaded PHBV nanofibers prepared by coaxial electrospinning were also analyzed by TEM (Figure 5).

As can be observed, ECO encapsulation within the nanofibers was confirmed at different locations throughout the membrane. Since the two components were electrospun from separate needles, inter-diffusion was minimized, preserving a well-defined core–shell architecture. This structural integrity ensured a clear physical separation between the core and the shell, which was essential when the goal was to protect the ECO.

Overall, the morphological analysis confirmed that electrospinning enabled the fabrication of PHBV-based nanofiber membranes with a homogeneous nanofiber distribution, forming an interconnected nanoporous network. Depending on the electrospinning configuration the extract was either blended with PHBV and incorporated into the nanofiber matrix or encapsulated within the nanofibers as the core of a core–shell structure.

The surface properties of the electrospun membranes were assessed through water contact angle measurements. This property is particularly relevant for packaging applications, where moisture resistance is a critical performance requirement [46]. For single electrospun membranes, WCA values of 106 ± 1°, 111 ± 4, 110 ± 1°, and 101 ± 1° were obtained for SPHBV, S8PHBV, S20PHBV and S30PHBV, respectively. The water contact angle remained relatively constant (around 110°) with increasing coffee oil content, suggesting that the incorporation of the oil did not substantially alter the overall surface wettability at low and intermediate concentrations. However, at the highest ECO content, WCA decreased to approximately 100°, which may be associated with the morphological changes observed by SEM, particularly the increased interactions and partial coalescence of the nanofibers. Membranes produced via coaxial electrospinning, presenting higher nanofiber diameters and, as discussed above, some regions of flattened nanofibers, showed lower WCA values, although the overall similar surface of the membranes was maintained. For coaxial systems, the addition of coffee extract significantly increased the contact angle from 82 ± 4° for CPHBV to 97 ± 4° for C15PHBV (one-way ANOVA, F(5, 30) = 81.09, *p* < 0.001). When coffee oil was used as the core, it provided internal support that preserved the cylindrical shape, as it was observed by TEM, and increased surface roughness.

**Figure 6:**
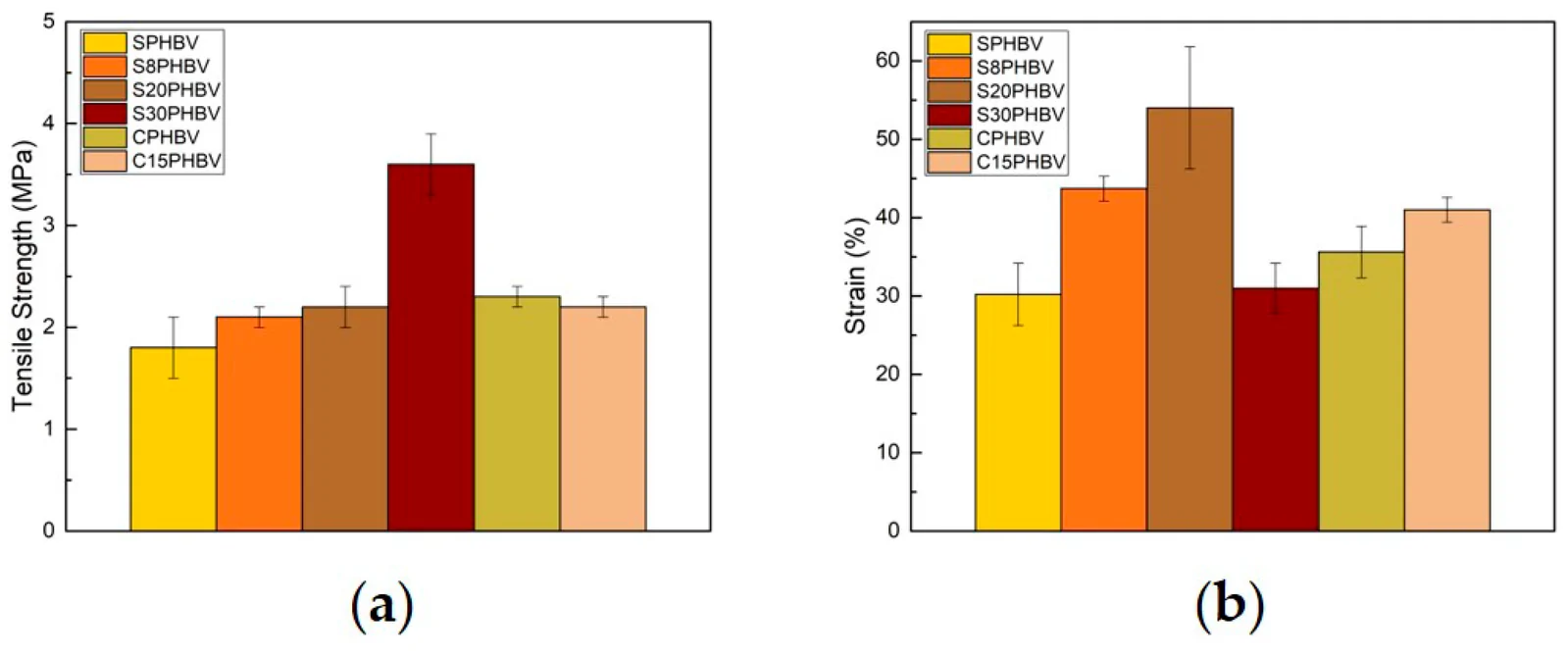
Effect of ECO content on the mechanical behavior of single and coaxial PHBV-based nanofiber membranes: tensile strength (**a**) and strain at break (**b**).

Finally, the mechanical behavior of the electrospun membranes was characterized using tensile tests. Figure S10 shows the stress–strain representative curve of each electrospun membrane. As reported in the literature, the mechanical properties of the electrospun membranes are influenced by several parameters, including the type of polymer used and the morphology of the nanofiber membrane [47]. The nanofiber diameter, the packing density of the nanofibers in the membrane and the interactions between nanofibers have been identified among key parameters [45,48]. As illustrated in Figure 6, the mechanical properties of the PHBV-based nanofiber membranes produced in the present study varied according to the electrospinning configuration and ECO content.

The tensile strength remained relatively constant in the low- and intermediate-ECO formulations produced using single and coaxial configurations. Tensile strength values of 1.8 ± 0.3, 2.1 ± 0.1 and 2.2 ± 0.2 MPa were measured for the SPHBV, S8PHBV and S20PHBV membranes electrospun in single configuration, respectively, and of 2.3 ± 0.1 and 2.2 ± 0.1 MPa for the CPHBV and C15PHBV membranes electrospun in coaxial configuration, respectively. However, the S30PHBV membrane, with the highest ECO content, exhibited a significantly higher tensile strength value of 3.6 ± 0.3 MPa (one-way ANOVA, F(5, 30) = 43.01, *p* < 0.001). Tukey’s HSD post hoc test identified significant differences between S30PHBV and SPHBV, S20PHBV, CPHBV, and C15PHBV, as well as between CPHBV and S20PHBV (*p* < 0.001). This improvement in mechanical strength may be related to the morphological differences observed in the SEM images. At higher ECO contents, increased inter-nanofiber junctions and partial nanofiber fusion have been observed. These features may promote stronger interactions within the fibrous network, contributing to the increase in tensile strength. Regarding strain at break, more pronounced differences were observed among all the formulations. For membranes electrospun using the single configuration, increasing the ECO content was found to result in higher deformation values, with strain increasing from 29% for the unloaded SPHBV membrane to 44% and 49% for the S8PHBV and S20PHBV membranes, respectively, which corresponds to a statistically significant difference (one-way ANOVA, F(5, 30) = 9.64, *p* < 0.001), with pairwise comparisons performed using Tukey’s post hoc test (*p* < 0.05). This behavior is probably due to an increase in polymer chain mobility, making the nanofibers more flexible and deformable before breaking. A similar trend has been reported for electrospun systems incorporating dispersed functional phases, where elongation at break increased while the structural integrity of the nanofibers was preserved [49]. However, when the concentration of ECO reached higher loadings, the deformation decreased markedly (to 29% for S30PHBV), indicating that the beneficial effect of ECO on membrane deformability was no longer maintained at such high loadings. The above mentioned partial nanofiber coalescence points observed at highest ECO content, may be responsible for the fiber mobility and deformation restriction, crumbling as the stress increased. Similar non-linear mechanical responses have been described for electrospun materials, where moderate levels of modification enhance flexibility, but higher degrees may reduce deformability [49,50]. This trend is consistent with previous observations in electrospun systems where natural oils act as mild plasticizers at low concentrations, but excess addition can reduce mechanical integrity, leading to non-linear mechanical behavior [51]. In the case of coaxial electrospun membranes, ECO increased the strain of the electrospun membranes from 31.5% for the CPHBV membrane to 40% for the C15PHBV membrane, while the tensile strength remained essentially unchanged. This behavior is consistent with previous studies on coaxial electrospun materials, which have shown that core–shell architectures can enhance functional performance whilst modifying deformation behavior, depending on the internal structure and the characteristics of the core–shell interface [16,52].

### 3.5. Assessment of the Activity of Electrospun Membranes

As has already been demonstrated, the phenolic compounds present in the ECO are characterized by their antioxidant activity, which is expected to be transferred to the electrospun membranes. With the aim of evaluating the antioxidant capacity of ECO in the obtained electrospun membranes, it was extracted from the membranes, and the DPPH inhibition percentage of the extractive solution was subsequently quantified following the procedure previously described. Table 2 summarizes the concentration of the extractive solutions, estimated using the calibration curve, as explained in Section 2.3, and their corresponding DPPH inhibition percentage.

It should be noted that the UV-based measurements generally overestimated the ECO concentrations compared to the theoretical values and exhibited considerable variability. Therefore, these results should be regarded as semiquantitative estimates rather than accurate measurements of the extracted ECO content. Nevertheless, the estimated concentrations followed a trend consistent with the theoretical ECO loading in the single electrospun membranes, providing useful comparative information. In the single configuration, all membranes exhibited inhibition percentages above 30%, which are comparable to the values reported in the literature for thymol and carvacrol, which are known to exhibit effective antioxidant activity in DPPH assays [53]. Although these differences were not statistically significant (*p* > 0.05), the observed trend suggests a potentially beneficial effect of increasing ECO content on the antioxidant performance of the membranes.

Interestingly, the highest mean inhibition percentage (44 ± 9%) was obtained for the coaxially obtained membrane (C15PHBV), which exhibited antioxidant activity comparable to that of S30PHBV (43 ± 3%), despite containing a lower theoretical ECO content. This result highlights the potential of coaxial electrospinning to produce antioxidant membranes with lower ECO loadings. Previous findings by Lan et al. [54] reported that coaxial nanofibers containing tea polyphenols provided sustained antioxidant release and offered greater protection to sensitive compounds against oxidation than conventional blend electrospinning. However, the present results do not provide direct evidence of a protective effect of the coaxial configuration on ECO stability. Therefore, further studies would be required to determine whether the coaxial configuration improves the stability and long-term preservation of the antioxidant compounds in the present system.

## 4. Conclusions

The extraction procedure was effective in obtaining coffee oil (ECO) with antioxidant activity and nearly balanced proportions of saturated and unsaturated fatty acids, predominantly palmitic and linoleic acids, respectively. Its antioxidant potential was confirmed by DPPH and ABTS radical scavenging assays and total phenolic content (TPC) analysis. ECO was successfully incorporated into electrospun PHBV nanofiber membranes, either blended with the polymer in the nanofiber or encapsulated in coaxial configuration, yielding bead-less nanofibers with homogeneous morphology. The presence of ECO shifted the maximum degradation temperature of the membranes towards higher temperatures, while TGA mass loss measurements provided estimates consistent with the theoretical ECO content. DSC thermograms of the membranes showed characteristic melting peaks for ECO and PHBV at the same temperatures as the pure components. FTIR spectra neither revealed significant shifts in the characteristic absorption bands nor the appearance of new signals. Overall, these findings suggest that both components largely retained their chemical identity and exhibited limited interactions during electrospinning. The ECO-loaded PHBV-based nanofiber membranes exhibited WCA values close to 100°. There were no significant differences in the WCA with respect to the amount of ECO, suggesting that surface wettability was primarily influenced by the nanofibrous structure rather than oil loading. The membranes demonstrated good mechanical performance, showing an increase in deformation at break and comparable tensile strength when low and intermediate ECO content was incorporated, while for the highest ECO content, mechanical behavior was governed by nanofibers structure. Regarding antioxidant activity, all ECO-loaded membranes exhibited radical scavenging capacity. Notably, coaxially electrospun membranes containing 15 wt% ECO showed DPPH inhibition values comparable to those of single-needle membranes containing 30 wt% ECO. These findings highlight the potential of coaxial electrospinning for developing antioxidant nanofibrous membranes with reduced ECO loading, although further studies are required to assess the possible influence of the core–shell structure on oil stability.

## Figures and Tables

**Table 1:** Antioxidant capacity results obtained for the extracted coffee oil.

DPPH (µmol TE g^−1^ ECO)	ABTS (µmol TE g^−1^ ECO)	TPC (mg GAE g^−1^ ECO)
82.34 ± 2.64	51.23 ± 1.31	22.05 ± 1.54

**Table 2:** Estimated amount of ECO-loaded electrospun nanofiber membranes and inhibition percentage of the extracted oil.

Membrane	UV-Estimated ECO Concentration in Membrane Extracts (mg/mL)	DPPH Inhibition %
S8PHBV	0.22 ± 0.14	31 ± 8
S20PHBV	0.32 ± 0.07	38 ± 1
S30PHBV	0.38 ± 0.11	43 ± 3
C15PHBV	0.26 ± 0.14	44 ± 9

## Data Availability

Data will be made available on request.

## Supplementary Materials

The following supporting information can be downloaded at https://www.mdpi.com/article/10.3390/polym18212780/s1. Figure S1: DPPH standard curve showing reaction absorbance as a function of Trolox concentration; Figure S2: ABTS standard curve showing reaction absorbance as a function of Trolox concentration; Figure S3: TPC standard curve showing absorbance as a function of gallic acid concentration; Figure S4: ECO calibration curve showing absorbance as a function of ECO concentration; Figure S5: SCAN-mode chromatogram of the ECO sample showing the major components. Figure S6: TG curve of ECO highlighting the weight loss of each degradation interval; Figure S7: TG curves of unloaded and ECO-loaded PHBV-based nanofiber membranes, highlighting the weight losses associated with the main components, with the first corresponding to PHBV and the second to ECO. The weight loss associated with ECO, relative to the weight loss experienced by neat ECO in this interval, was used to experimentally estimate the ECO content; Figure S8: FTIR spectra of ECO highlighting the characteristic vibration bands; Figure S9: Fiber diameter distribution histograms of the electrospun membranes.; Table S1: Melting enthalpy (ΔHm) of ECO around 5 °C measured by DSC for neat ECO and S8PHBV, S20PHBV, S30PHBV and C15PHBV membranes obtained by single and coaxial electrospinning; Figure S10: Stress–strain curves of SPHBV, S8PHBV, S20PHBV, S30PHBV, CPHBV and C15PHBV.

## Abbreviations

The following abbreviations are used in this manuscript:

ABTS	2,2′-Azino-bis(3-ethylbenzothiazoline-6-sulfonic acid)
ATR	Attenuated Total Reflectance
DCM	Dichloromethane
DMSO	Dimethyl Sulfoxide
DPPH	2,2-Diphenyl-1-picrylhydrazyl
DSC	Differential Scanning Calorimetry
DTG	Derivative Thermogravimetry
ECO	Extracted Coffee Oil
FC	Folin-Ciocalteau
FTIR	Fourier Transform Infrared Spectroscopy
NaOH	Sodium Hydroxide
PHAs	Polyhydroxyalkanoates
PHBV	Poly(3-hydroxybutyrate-co-3-hydroxyvalerate)
SCG	Spent Coffee Grounds
SEM	Scanning Electron Microscopy
TFE	Trifluoroethanol
TEM	Transmission Electron Microscopy
TG	Thermogravimetry
TGA	Thermogravimetric Analysis
TPC	Total Phenolic Content
WCA	Water Contact Angle
