# Supplementary material for: Active Poly(3-hydroxybutyrate-co-3-hydroxyvalerate) Electrospun Membranes Incorporating Antioxidant Oil from Spent Coffee Grounds
Source: Polymers (Basel). 2026 Nov 7;18(21):2780. doi: 10.3390/polym18212780 (PMC13765655; doi:10.3390/polym18212780)
Supplement: Supplementary file 1 [file polymers-18-02780-s001.zip › polymers-4600253-supplementary.pdf]

Supporting Information

Active Poly(3-hydroxybutyrate-co-3-hydroxyvalerate) Electrospun Membranes Incorporating Antioxidant Oil from Spent Coffee Grounds

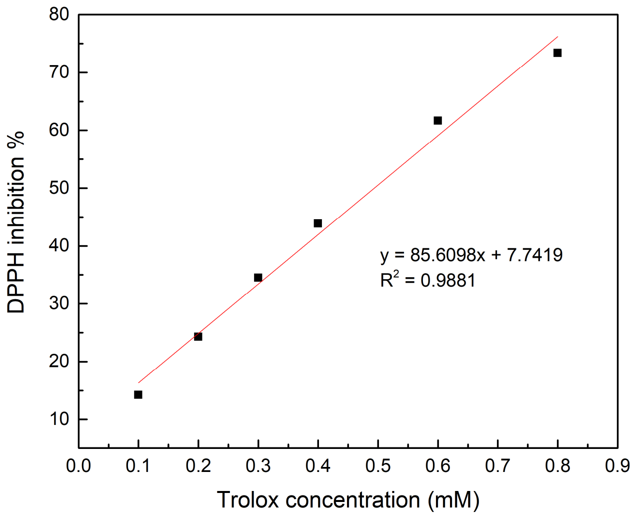

Figure S1. DPPH standard curve showing reaction absorbance as a function of Trolox concentration.

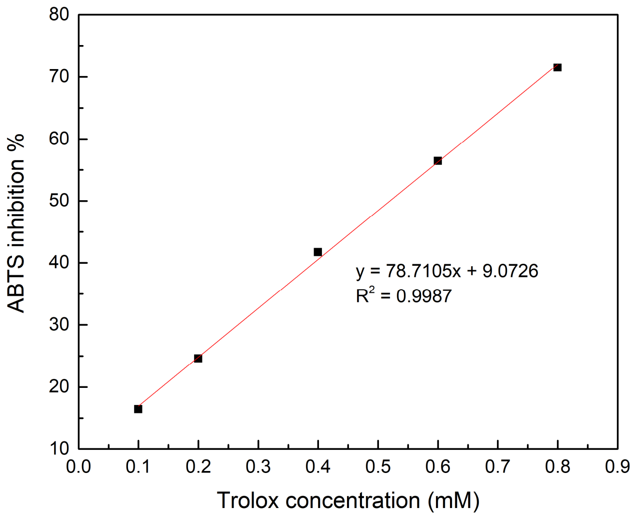

Figure S2. ABTS standard curve showing reaction absorbance as a function of Trolox concentration.

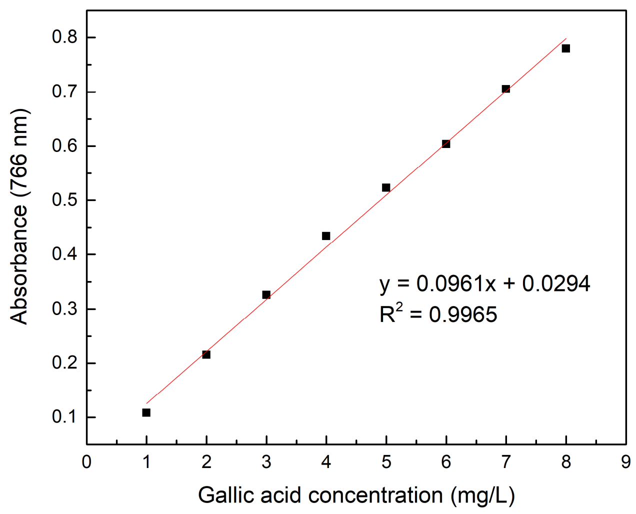

Figure S3. TPC standard curve showing absorbance as a function of gallic acid concentration.

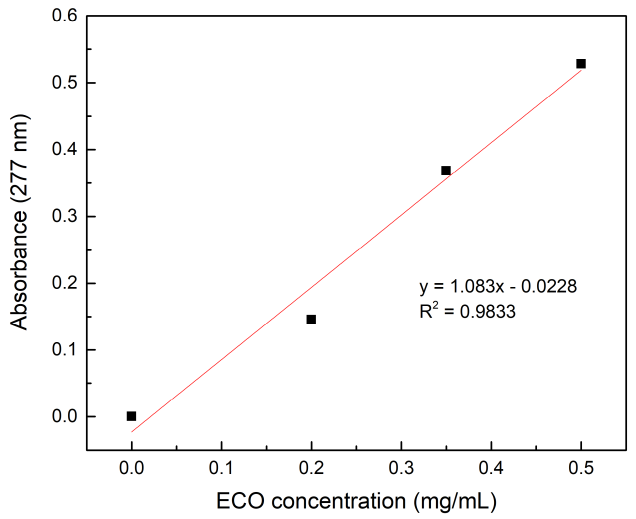

Figure S4. ECO calibration curve showing absorbance as a function of ECO concentration.

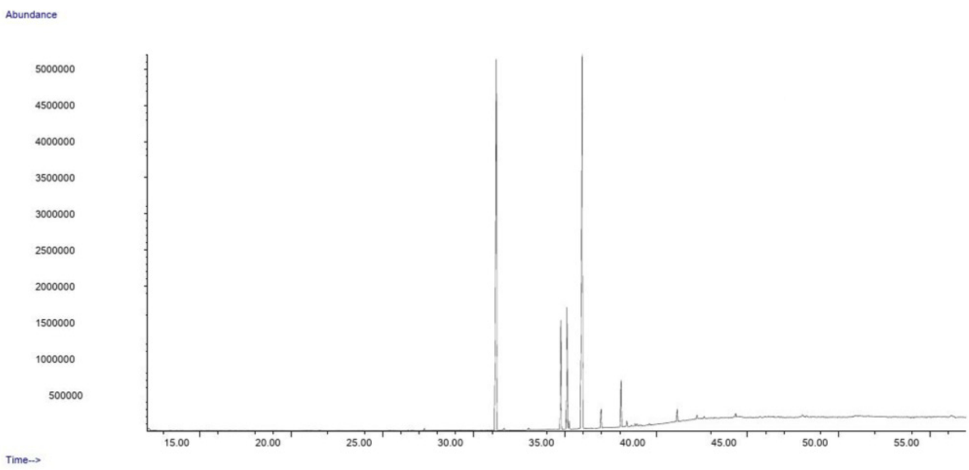

Figure S5. SCAN-mode chromatogram of the ECO sample showing the major components.

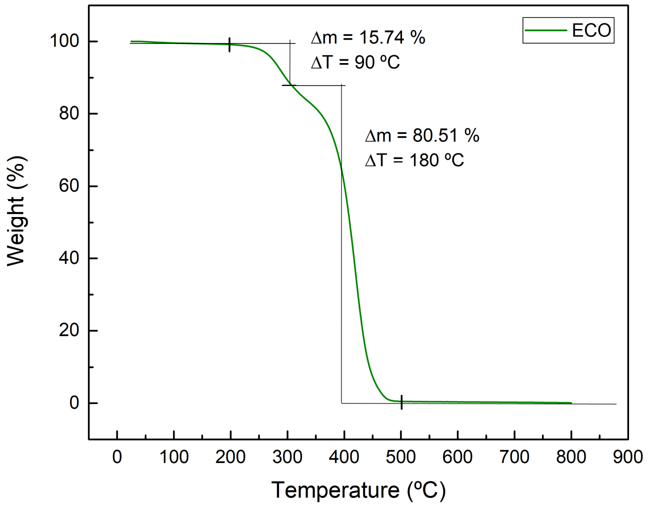

Figure S6. TG curve of ECO highlighting the weight loss of each degradation interval.

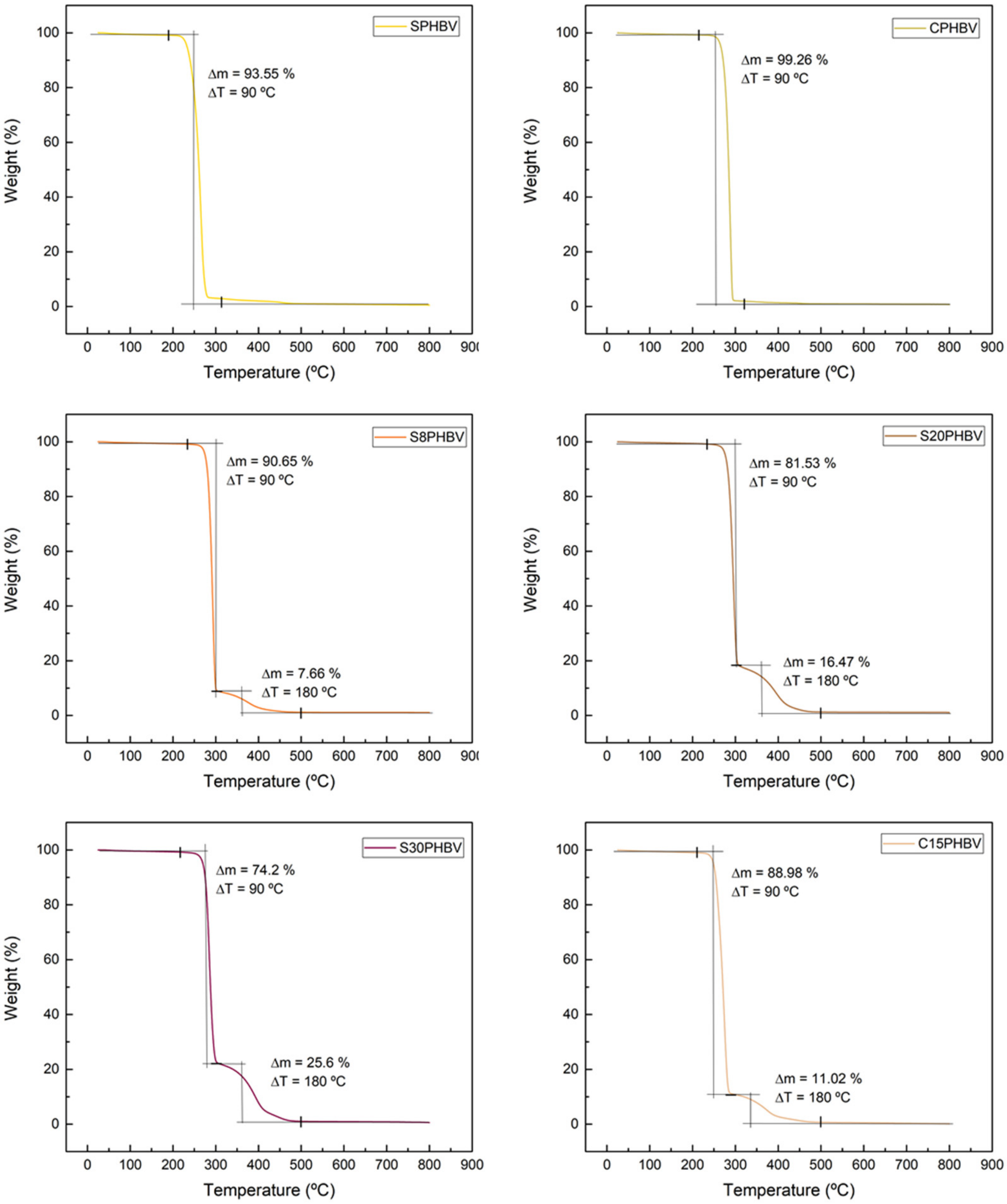

Figure S7. TG curves of unloaded and ECO-loaded PHBV-based nanofiber membranes, highlighting the weight losses associated with the main components, with the first corresponding to PHBV and the second to ECO. The weight loss associated with ECO, relative to the weight loss experienced by neat ECO in this interval, was used to experimentally estimate the ECO content.

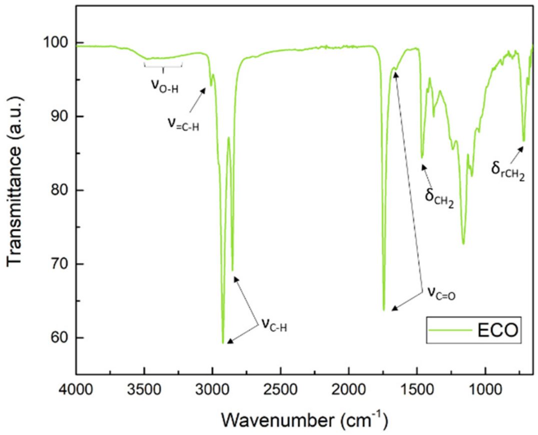

Figure S8. FTIR spectra of ECO highlighting the characteristic vibration bands.

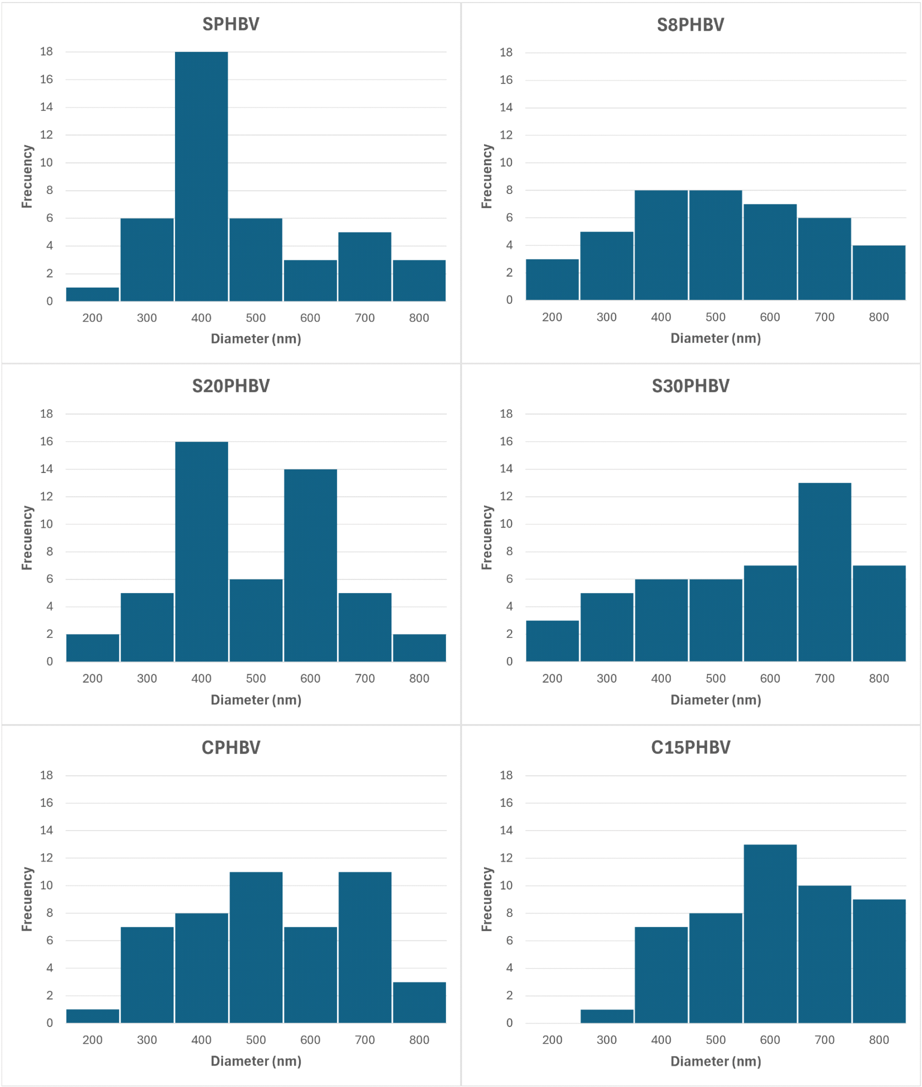

Figure S9. Fiber diameter distribution histograms of the electrospun membranes.

Table S1. Melting enthalpy (ΔH_m) of ECO around 5 ºC measured by DSC thermogram of neat ECO, and S8PHBV, S20PHBV, S30PHBV and C15PHBV membranes, obtained both by single and coaxial electrospinning.

**Sample**	**ΔH_m (J/g)**
ECO	39.0
S8PHBV	1.8
S20PHBV	6.7
S30PHBV	10.4
C15PHBV	2.5

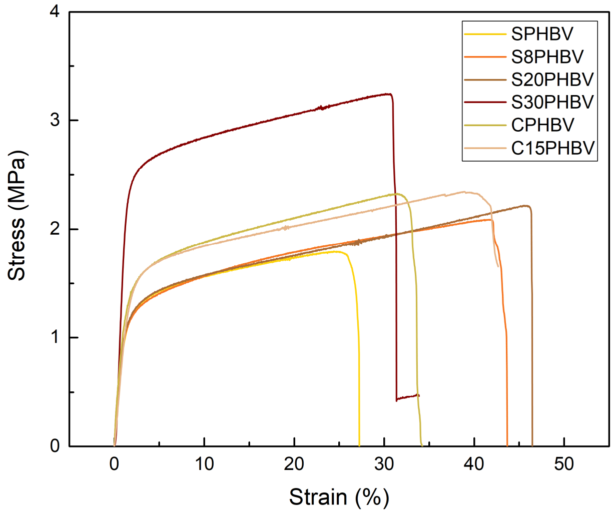

Figure S10. Stress-strain curves of SPHBV, S8PHBV, S20PHBV, S30PHBV, CPHBV and C15PHBV.
